# Diagnostic Accuracy and Time Efficiency of Artificial Intelligence for Intracranial Hemorrhage Detection on Emergency Brain CT

**DOI:** 10.3390/diagnostics16142219

**Published:** 2026-07-16

**Authors:** Ömer Damar, Mahmut Yaman, Mehmet Tahir Gokdemir

**Affiliations:** 1 Department of Emergency Medicine, Faculty of Medicine, Mardin Artuklu University, 47200 Mardin, Turkey; tahirgokdemir@artuklu.edu.tr; 2 Department of Emergency Medicine, Faculty of Medicine, Dicle University, 21280 Diyarbakır, Turkey; drmahmutyaman@hotmail.com

**Keywords:** intracranial hemorrhage, artificial intelligence, brain computed tomography, emergency department, diagnostic accuracy

## Abstract

**Background**: Intracranial hemorrhage (ICH) is a neurological emergency associated with high mortality and morbidity, requiring rapid diagnosis and early intervention. Artificial intelligence (AI)-based imaging systems have recently gained attention as supportive tools for accelerating emergency brain computed tomography (CT) interpretation. This study aimed to evaluate the diagnostic accuracy and time efficiency of an AI system for detecting ICH on emergency brain CT scans. **Methods**: This retrospective study included 375 patients who underwent non-contrast brain CT in the emergency department. AI-based image analysis results were compared with final radiologist reports accepted as the reference standard. Sensitivity, specificity, predictive values, accuracy, area under the curve (AUC), and Cohen’s kappa were calculated. Interpretation times, subgroup analyses according to presentation type and work shifts, and hemorrhage volume correlations between AI and the ABC/2 method were also evaluated. **Results**: Intracranial hemorrhage was detected in 235 patients (62.7%). The AI system achieved a sensitivity of 94.0%, specificity of 87.9%, positive predictive value of 92.9%, negative predictive value of 89.8%, and overall accuracy of 91.7%. The AUC was 0.909, with a Cohen’s kappa coefficient of 0.823. Mean interpretation time was significantly shorter for AI compared with radiologists (11.9 ± 3.0 vs. 70.9 ± 25.4 min, *p* < 0.001). AI-derived hemorrhage volume measurements strongly correlated with the ABC/2 method (rho = 0.887, *p* < 0.001). **Conclusions**: The AI system demonstrated high diagnostic accuracy and a significant reduction in interpretation time for detecting intracranial hemorrhage on emergency brain CT, supporting its potential role as a decision-support tool in emergency radiology workflows.

## 1. Introduction

Intracranial hemorrhages are among the neurological emergencies that require rapid diagnosis and early treatment in the emergency department and are associated with significant clinical outcomes in terms of mortality and morbidity. In particular, spontaneous intracranial hemorrhages, traumatic brain injuries, and anticoagulant-related hemorrhages represent clinical conditions in which early imaging evaluation is critically important [1,2]. Brain computed tomography (CT) is widely accepted as the first-line imaging modality for the evaluation of acute intracranial hemorrhage [3]. Increasing patient volume in emergency departments, the marked rise in imaging requests, and the growing radiology workload may lead to prolonged CT reporting times [4]. Delays in radiological evaluation processes have been reported more frequently, particularly during night shifts, periods of high patient flow, and situations involving limited human resources [5]. Early detection of intracranial hemorrhage is of major clinical importance for timely neurosurgical intervention, intensive care planning, and treatment strategies aimed at reducing mortality [6]. Therefore, interest in rapid decision-support systems in emergency radiology practice has increased in recent years.

Artificial intelligence (AI)-based imaging analysis systems have become increasingly utilized in the field of neurological imaging, particularly following advances in deep learning algorithms [7]. AI systems developed for the automated detection of intracranial hemorrhage on brain CT images have been reported to demonstrate high sensitivity and specificity [8]. However, a substantial proportion of existing studies have primarily focused on diagnostic accuracy, whereas parameters such as interpretation time, inter-shift performance variability, and contribution to clinical workflow have been evaluated to a more limited extent [9]. Furthermore, the clinical applicability of advanced analyses, including the classification of intracranial hemorrhage subtypes and automated hemorrhage volume measurement, remains under investigation. In the emergency department setting, AI-supported systems are considered valuable adjunctive tools for rapid preliminary assessment, particularly for supporting radiology reporting workflows and facilitating the early identification of critically ill patients during periods of high patient volume [10]. Nevertheless, clinical studies evaluating the real-world performance of AI systems remain limited.

In this study, we aimed to compare the diagnostic performance of an AI system with radiologist evaluation for the detection of intracranial hemorrhage on brain CT images obtained in the emergency department. The primary objective of the study was to evaluate the sensitivity, specificity, accuracy, and level of agreement between the AI system and radiologist assessment in detecting intracranial hemorrhage. Secondary objectives included comparison of interpretation times, investigation of performance variability across different presentation types and work shifts, and evaluation of the correlation between hemorrhage volume calculated by the AI system and the ABC/2 method. The novelty of the present study lies in its comprehensive real-world evaluation of a commercially available AI platform across multiple clinically relevant dimensions simultaneously—including binary and categorical diagnostic accuracy, hemorrhage subtype classification, volumetric correlation, interpretation time efficiency, and performance variability across presentation types and work shifts—within an unselected emergency department patient cohort. Unlike prior validation studies that have largely focused on isolated performance metrics in controlled datasets, this study provides an integrated analysis that reflects actual clinical deployment conditions, thereby offering practical insights into the operational value and limitations of AI-assisted intracranial hemorrhage detection as a decision-support tool in emergency radiology workflows.

## 2. Materials & Methods

### 2.1. Study Design and Setting

This retrospective diagnostic accuracy study was conducted to evaluate brain computed tomography (CT) images obtained in the emergency department for suspected intracranial hemorrhage. In the study, the diagnostic performance and interpretation time of an artificial intelligence (AI)-based image analysis system were compared with radiologist reports accepted as the reference standard. Non-contrast emergency brain CT examinations were evaluated, and both binary classification (hemorrhage present/absent) and category-based hemorrhage classification analyses were performed.

### 2.2. Participant Selection

A total of 375 consecutive patients undergoing emergency non-contrast brain CT for suspected acute intracranial pathology between January 2025 and December 2025 were retrospectively included in the study. According to the reason for presentation, patients were classified into neurological presentations and trauma presentations. In addition, evaluations were analyzed separately according to day, evening, and night shifts. Inclusion criteria were defined as having undergone non-contrast brain CT in the emergency department, availability of AI analysis output, and completion of the radiology report. Patients with inadequate image quality for evaluation, cases with incomplete data, and examinations in which AI analysis could not be performed due to technical reasons were excluded from the study. Data were collected from the Emergency Department of Mardin Artuklu University Faculty of Medicine, a tertiary-care referral center located in Mardin, Turkiye, serving a predominantly adult mixed neurological and trauma patient population.

### 2.3. Imaging Protocol and Artificial Intelligence System

All non-contrast brain CT examinations were acquired using a standardized institutional emergency imaging protocol on a 16-slice multidetector CT scanner (Toshiba Aquilion, Canon Medical Systems Corporation, Otawara, Japan). Imaging parameters included 120 kVp tube voltage, automatic tube current modulation ranging between 100–300 mAs, axial image reconstruction at 5 mm slice thickness, additional 0.5 mm thin-section reconstruction, and iterative reconstruction algorithms for image optimization. Multiplanar reformatted images were generated when clinically indicated. The AI system used in the study was a commercially available deep learning-based clinical decision-support platform (hStroke CT module, Hevi AI^®^, Hevi AI Health Technologies Inc., Istanbul, Türkiye; available at: www.heviai.com) integrated into the institutional PACS workflow for automated analysis of DICOM-format non-contrast brain CT images. The AI system automatically performed exam-level analysis of non-contrast brain CT images and generated outputs regarding intracranial hemorrhage detection, hemorrhage subtype classification, interpretation time, and subtype-specific volumetric measurements including intraparenchymal hemorrhage (IPH), isolated intraventricular hemorrhage (IVH), subdural hematoma (SDH), and epidural hematoma (EDH) volumes. The software was integrated into the institutional emergency radiology workflow as a diagnostic decision-support tool. AI-generated results were compared with final signed radiology reports prepared by board-certified radiologists, which were accepted as the reference standard. Radiologists were blinded to the AI-generated outputs, and radiological evaluations were performed independently from the AI system. Regarding the technical infrastructure of the evaluated AI system: the hStroke CT module (Hevi AI^®^) is a convolutional neural network (CNN)-based deep learning system specifically designed for automated detection and classification of intracranial hemorrhage on non-contrast brain CT images. The system employs a multi-stage detection pipeline consisting of slice-level feature extraction using a deep residual network (ResNet) architecture, followed by exam-level aggregation and hemorrhage subtype classification. The model was trained on a large multicenter dataset of annotated non-contrast brain CT scans and underwent internal validation prior to commercialization; however, detailed architecture parameters, training dataset size, and specific validation metrics are proprietary and have not been publicly disclosed by the manufacturer. The system operates in a fully automated, end-to-end fashion without requiring manual input, and generates binary hemorrhage classification outputs alongside subtype-specific probability scores and volumetric estimates. It is acknowledged that the limited transparency inherent to this black-box commercial system restricts the ability to fully assess potential biases, generalizability, or failure modes, which represents a limitation of this evaluation as discussed in the Section 5. The reference standard radiological reports were prepared by board-certified diagnostic radiologists with subspecialty training in neuroradiology. All reporting radiologists had a minimum of five years of post-residency experience in neuroimaging, and emergency brain CT reporting was performed exclusively by this group. Emergency radiologists or trainees did not serve as independent report authors for this study. A total of three neuroradiology-trained radiologists participated in the reporting process across the study period; each examination was interpreted by a single on-duty radiologist, and the final signed report constituted the reference standard for that case. In cases where the clinical team raised concern about an initial report during the study period, a second senior radiologist review was performed and the revised report was used as the reference standard. Discrepant or uncertain cases that could not be resolved by senior radiologist review were excluded from the study per the pre-specified exclusion criteria (incomplete data). Regarding blinding, although the AI system is integrated into the institutional PACS workflow, AI-generated outputs were not displayed to reporting radiologists during their evaluation. AI outputs were recorded separately by the research team after radiologist reporting was finalized, and radiologists were explicitly instructed not to access AI outputs during interpretation, thereby ensuring complete blinding.

Intracranial hemorrhage subtypes were classified as no hemorrhage, IPH, SDH, IPH + IVH, IVH, and EDH. In the binary classification analysis, all hemorrhage subtypes were included within the hemorrhage-positive group.

### 2.4. Measurements and Outcomes

Age, sex, triage score, presentation type, presence of hemorrhage, hemorrhage subtype, shift information, AI interpretation time, and radiologist interpretation time were recorded. The primary outcome was the diagnostic performance of the AI system in detecting intracranial hemorrhage. Secondary outcomes included category-based diagnostic agreement, differences in interpretation times, subgroup performances according to presentation type and work shift, and the correlation between hemorrhage volume calculated by the AI system and the ABC/2 method. In hemorrhagic cases, hemorrhage volume was calculated using both the AI system and the ABC/2 formula. The ABC/2 method was calculated using the formula A × B × C/2, where A represented the largest hemorrhage diameter, B represented the diameter perpendicular to A, and C represented the depth obtained by multiplying the number of slices containing hemorrhage by the slice thickness. Radiologist interpretation time was defined as the total report turnaround time—the interval between CT image availability in the institutional PACS system and the timestamp of the final signed radiology report—automatically extracted from PACS log data. This measure encompasses image review, dictation, and finalization processes and should not be interpreted as the time spent solely on image viewing. AI interpretation time was defined as the interval between DICOM image upload to the AI system and generation of the automated output report, as recorded by the platform’s internal timestamp log. It is acknowledged that these two time metrics are not directly equivalent: AI processing time reflects pure computational analysis time, whereas radiologist turnaround time additionally incorporates queue wait time, competing clinical workload, report dictation, and administrative finalization steps. This methodological difference is discussed as a limitation of the time-efficiency comparison (see Section 5).

### 2.5. Ethical Approval

This study was conducted in accordance with the principles of the Declaration of Helsinki. Ethical approval was obtained from the Non-Interventional Clinical Research Ethics Committee of Mardin Artuklu University Faculty of Medicine (approval date: 22 April 2025; decision number: 2025/4-40). All patient data were anonymized and analyzed solely for scientific purposes. Due to the retrospective nature of the study, the requirement for informed consent was waived by the ethics committee.

### 2.6. Statistical Analysis

Statistical analyses were performed using IBM SPSS Statistics version 26, IBM Corp., Armonk, NY, USA. Continuous variables were expressed as mean ± standard deviation or median (interquartile range), whereas categorical variables were presented as numbers and percentages. The distribution of continuous variables was assessed using appropriate normality tests and visual methods. The Mann–Whitney U test was used for comparisons of continuous variables between two independent groups. A paired non-parametric Wilcoxon signed-rank test was applied to compare AI and radiologist interpretation times. The Kruskal–Wallis test was used for inter-shift comparisons. Categorical variables were analyzed using the chi-square test or Fisher’s exact test when appropriate.

Diagnostic performance analyses included the calculation of sensitivity, specificity, positive predictive value, negative predictive value, accuracy, F1 score, Matthews correlation coefficient, positive likelihood ratio, and negative likelihood ratio. Receiver operating characteristic (ROC) curve analysis was performed, and the area under the curve (AUC) was reported with 95% confidence intervals (CIs). Bootstrap resampling with 2000 iterations was used to calculate AUC confidence intervals.

Agreement between the AI system and the reference standard radiological evaluation was assessed using Cohen’s kappa coefficient. Quadratic weighted kappa analysis was applied for category-based evaluations. Subgroup analyses were performed according to presentation type and work shift. The relationship between hemorrhage volume calculated by the AI system and the ABC/2 method was evaluated using Spearman correlation analysis. A *p* value of <0.05 was considered statistically significant.

## 3. Results

A total of 375 patients were included in the study. The mean age of the patients was 52.8 ± 20.8 years, and intracranial hemorrhage was detected in 235 patients (62.7%). The mean age of patients with hemorrhage was significantly higher than that of patients without hemorrhage (57.4 ± 19.8 vs. 45.2 ± 20.1 years, *p* < 0.001). Overall, 54.1% of the patients were male, and sex distribution did not differ significantly according to the presence of hemorrhage (*p* = 0.238). The mean triage score was calculated as 2.9 ± 1.1. Patients with hemorrhage had significantly lower triage scores compared with those without hemorrhage (2.4 ± 0.9 vs. 3.1 ± 1.1, *p* < 0.001). Regarding presentation type, neurological presentations were more frequent in the hemorrhage group (64.7% vs. 41.4%), whereas trauma presentations were more common in the non-hemorrhage group (58.6% vs. 35.3) (Table 1).

The mean interpretation time of the AI system was 11.9 ± 3.0 min, whereas the mean radiologist interpretation time was 70.9 ± 25.4 min. Median interpretation times were 12.0 min (IQR: 9.6–14.0) and 69.8 min (IQR: 50.9–89.6), respectively. The minimum–maximum interpretation time ranged between 4.6 and 18.6 min for the AI system and between 28 and 115 min for radiologists. The mean time difference between the two methods was 59.0 min, and this difference was statistically significant (*p* < 0.001). In the shift-based analysis, the mean interpretation times of the AI system during day, evening, and night shifts were 11.8, 12.1, and 11.9 min, respectively. Corresponding radiologist interpretation times were 69.4, 71.8, and 72.0 min. No significant difference in interpretation times was observed between shifts (*p* > 0.05) (Table 2).

**Figure 1:**
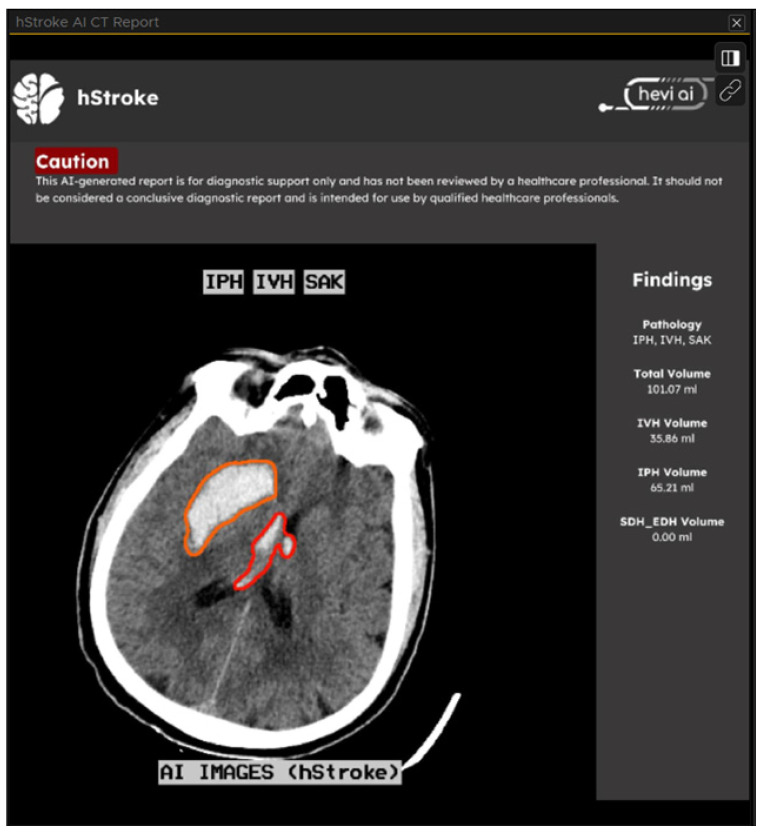
True positive case: AI system (hStroke CT module, Hevi AI^®^) correctly detected and segmented intraparenchymal hemorrhage (IPH) with concomitant intraventricular hemorrhage (IVH) and subarachnoid extension (SAK) on non-contrast emergency brain CT. The AI-generated segmentation overlay (orange contour = IPH; red contour = IVH) accurately delineates the hemorrhagic lesion. Findings panel shows total hemorrhage volume of 101.07 mL, IVH volume of 35.86 mL, IPH volume of 65.21 mL, and SDH/EDH volume of 0.00 mL. CT = computed tomography; AI = artificial intelligence; IPH = intraparenchymal hemorrhage; IVH = intraventricular hemorrhage; SAK = subarachnoid hemorrhage.

**Figure 2:**
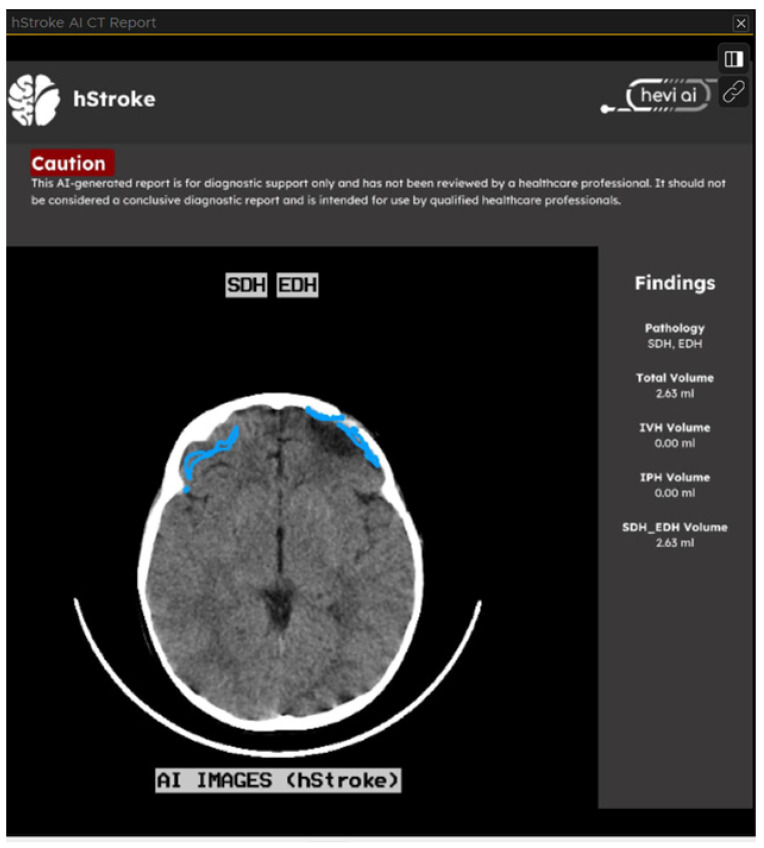
False positive case: AI system incorrectly classified bilateral frontoparietal subdural/epidural hyperdense collections (SDH/EDH) with a total detected volume of 2.63 mL on non-contrast emergency brain CT. The AI-generated segmentation overlay (blue contour) highlights bilateral convexity regions that were identified as SDH/EDH by the algorithm; however, the reference standard radiologist report did not confirm pathological hemorrhage at this location, attributing the finding to physiological or artifactual hyperdensity. This case illustrates a characteristic false-positive pattern involving thin extra-axial hyperdensities near the calvarium. CT = computed tomography; AI = artificial intelligence; SDH = subdural hematoma; EDH = epidural hematoma.

In the binary classification analysis, the AI system classified 221 patients as true positive, 123 as true negative, 17 as false positive, and 14 as false negative. The sensitivity of the AI system was 94.0% (95% CI: 91.2–96.2), specificity was 87.9% (95% CI: 84.2–90.9), positive predictive value was 92.9% (95% CI: 90.0–95.1), and negative predictive value was 89.8% (95% CI: 86.3–92.5). Overall accuracy was 91.7% (95% CI: 88.6–94.1). The F1 score was 0.935, Matthews correlation coefficient was 0.823, and Cohen’s kappa coefficient was 0.823. The area under the ROC curve was 0.909 (95% CI: 0.875–0.939). The positive likelihood ratio was 7.76, whereas the negative likelihood ratio was 0.068 (Table 3). Among the 14 false-negative cases, the most common missed hemorrhage subtypes were isolated intraventricular hemorrhage (IVH; *n* = 5, 35.7%), followed by small-volume subdural hematoma (SDH; *n* = 4, 28.6%), intraparenchymal hemorrhage with intraventricular hemorrhage (IPH + IVH; *n* = 3, 21.4%), and epidural hematoma (EDH; *n* = 2, 14.3%). Missed IVH cases predominantly involved small-volume periventricular blood, and missed SDH cases were characterized by thin layering collections with maximum thickness below 5 mm. These findings suggest that the AI system’s false-negative rate was primarily associated with small lesion volumes and anatomically subtle hemorrhage presentations. Among the 17 false-positive cases, the most common patterns included symmetric physiological hyperdensity misclassified as intraparenchymal hemorrhage (*n* = 6, 35.3%), beam-hardening and motion artifacts mimicking extra-axial hemorrhage (*n* = 5, 29.4%), dense dural calcifications misidentified as subdural collections (*n* = 4, 23.5%), and other technical or anatomical confounders (*n* = 2, 11.8%). Representative AI system output examples—including cases of true positive hemorrhage detection, false-positive artifact misclassification, and false-negative small-volume IVH—are depicted in Figure 1 and Figure 2 (supplementary visual output), illustrating the AI detection overlay on non-contrast brain CT images, the subtype classification label, and the associated probability score generated by the system for each case category. These qualitative examples highlight both the operational strengths and characteristic failure modes of the evaluated platform in a real-world emergency setting.

In the categorical analysis, exact agreement between the AI system and the reference standard radiological evaluation was observed in 90.1% of cases, and the quadratic weighted Cohen’s kappa coefficient was calculated as 0.892 (95% CI: 0.863–0.921). In subtype classification, correct classification rates ranged from 84.6% to 92.9%, whereas category-based Cohen’s kappa coefficients ranged between 0.712 and 0.841 (Table 4).

**Figure 3:**
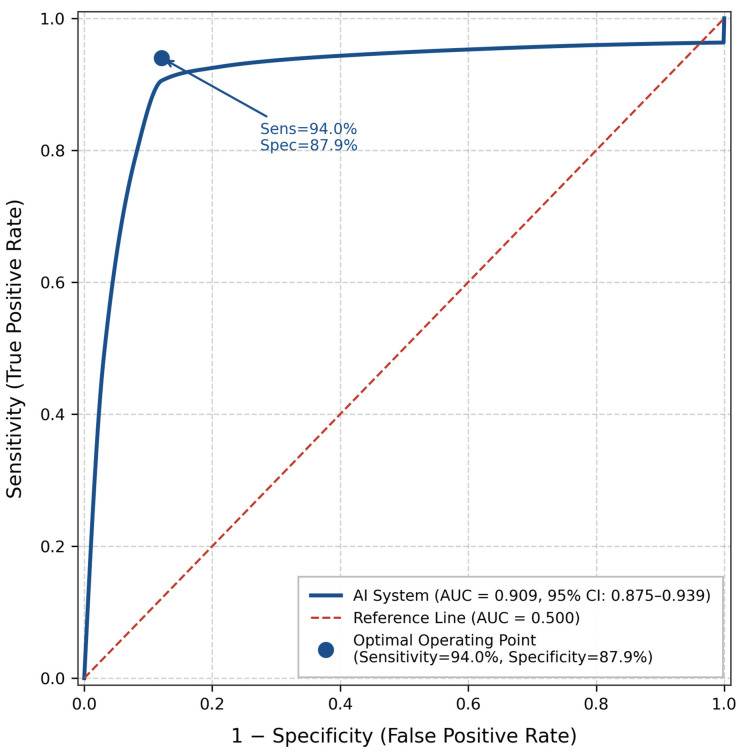
Receiver operating characteristic (ROC) curve for the AI system in the detection of intracranial hemorrhage on emergency brain CT. The area under the curve (AUC) was 0.909 (95% CI: 0.875–0.939). The optimal operating point is indicated (sensitivity = 94.0%, specificity = 87.9%). CI: Confidence interval.

In neurological presentations, sensitivity was 94.8% (95% CI: 90.9–97.1), specificity was 86.7% (95% CI: 81.4–90.6), accuracy was 91.4%, AUC was 0.952, and Cohen’s kappa coefficient was 0.843. In trauma presentations, sensitivity was 87.1% (95% CI: 81.1–91.4), specificity was 94.2% (95% CI: 89.5–96.9), accuracy was 90.3%, AUC was 0.901, and Cohen’s kappa coefficient was 0.798. Differences between the groups were statistically significant (*p* = 0.011). In the shift-based analysis, sensitivity, specificity, accuracy, and AUC during the day shift were 94.8%, 91.2%, 93.4%, and 0.962, respectively. During the evening shift, sensitivity was 93.3%, specificity was 89.7%, accuracy was 92.0%, and AUC was 0.941. During the night shift, sensitivity was 91.4%, specificity was 87.1%, accuracy was 89.6%, and AUC was 0.908. Cohen’s kappa coefficients during day, evening, and night shifts were 0.856, 0.831, and 0.789, respectively. The overall difference between shifts was statistically significant (*p* = 0.031). In hemorrhagic patients, the mean hemorrhage volume calculated by the AI system was 28.4 ± 18.7 mL, whereas the mean volume calculated using the ABC/2 formula was 29.1 ± 17.3 mL. The Spearman correlation coefficient between the AI system and the ABC/2 method was rho = 0.887 (*p* < 0.001) (Table 5) (Figure 3).

## 4. Discussion

In this study, the artificial intelligence (AI) system demonstrated high accuracy in detecting intracranial hemorrhage on brain CT examinations performed in the emergency department, provided substantially shorter interpretation times compared with radiologist evaluation, and showed strong agreement in both hemorrhage subtype classification and hemorrhage volume estimation. In addition, performance was not completely homogeneous across all clinical conditions; sensitivity was higher in neurological presentations, whereas specificity was higher in trauma presentations, and a relative decline in diagnostic performance was observed during night shifts.

The approximately 59 min difference in interpretation time between the AI system and radiologist evaluation observed in our study represents not only a statistically significant finding but also a clinically important one. Early diagnosis in suspected intracranial hemorrhage is known to directly influence critical treatment steps, including neurosurgical evaluation, intensive care planning, and anticoagulant reversal [4,11]. Boland et al. identified radiology report turnaround time as one of the major determinants of emergency department workflow, whereas Hanna et al. demonstrated an association between prolonged shifts, high imaging volume, and interpretation errors [4,5]. Therefore, the observed time gain reflects not only the speed of the algorithm but also a potentially clinically meaningful advantage, as it may facilitate earlier physician notification and triage prioritization; however, direct evidence of improved patient outcomes was not assessed in this study and remains to be demonstrated in prospective clinical trials. Similarly, Arbabshirani et al. demonstrated that AI-assisted prioritization systems could significantly reduce median diagnostic time [9]. Titano et al. also reported that deep learning–based systems may accelerate workflow in the early detection of acute neurological events [10]. However, findings in the literature are not entirely consistent. In a prospective real-world study, Savage et al. showed that an AI-supported triage system did not significantly improve radiologist performance or report turnaround time [12]. This suggests that the clinical contribution of AI systems may depend not only on algorithmic performance but also on the manner in which these systems are integrated into local clinical workflows [9,12,13]. An important methodological caveat is that the time metrics compared in our study are not strictly equivalent: AI interpretation time represents pure computational processing time, whereas radiologist interpretation time reflects total report turnaround time inclusive of queue delay, competing workload, dictation, and report finalization. This asymmetry means that the observed 59 min difference should not be interpreted as the time a radiologist requires to read a single brain CT image, but rather as the difference in time-to-report-availability at the system level—a clinically relevant distinction for emergency workflow prioritization, but one that overestimates the pure reading time advantage of AI over a focused radiologist review. This limitation is explicitly acknowledged in the Section 5.

From a diagnostic performance perspective, the sensitivity of 94.0%, specificity of 87.9%, accuracy of 91.7%, and AUC value of 0.909 obtained in our study are consistent with the current literature. Chilamkurthy et al. reported that deep learning algorithms were able to detect critical findings on head CT examinations with high accuracy [8]. Lee et al. demonstrated that explainable AI models could achieve performance comparable to expert radiologists, while Wang et al. showed that high AUC values could be maintained even in external validation datasets [14,15]. On a broader scale, the systematic review and meta-analysis conducted by Jørgensen et al. reported that CNN-based systems were largely comparable to radiologist performance in retrospective series, although performance could partially decline in external validation datasets [16]. Similarly, Maghami et al. reported a pooled sensitivity of 0.917 and a pooled specificity of 0.945, whereas Karamian and Seifi demonstrated in their 2025 meta-analysis that deep learning exhibited strong diagnostic performance for non-contrast brain CT examinations [17,18]. Nevertheless, Agarwal et al. emphasized that the current evidence remains insufficient for routine clinical integration [19]. Our findings likewise suggest that AI may be more appropriately positioned as a decision-support tool that supports radiologist workflow and facilitates prioritization rather than as an independent diagnostic system. In particular, the low negative likelihood ratio indicates that negative AI results may have clinical value in excluding intracranial hemorrhage. It is important to acknowledge that the diagnostic performance reported in this study is specific to the evaluated commercial platform (hStroke CT module, Hevi AI^®^) and may not be generalized to other AI systems utilizing different deep learning architectures, training datasets, or image preprocessing pipelines. Performance characteristics of AI systems for intracranial hemorrhage detection vary considerably across platforms, as demonstrated by published comparative evaluations. Therefore, institutional validation prior to clinical deployment of any AI system is strongly recommended.

The high categorical agreement and strong weighted kappa value observed in the subtype analyses indicate that the AI system was able not only to detect the presence of hemorrhage but also to successfully classify hemorrhage subtypes. However, the relatively lower performance observed in isolated IVH and combined IPH + IVH patterns may be related to the difficulty of evaluating small ventricular foci and the anatomical complexity of mixed hemorrhage patterns. These findings are consistent with the existing literature. Lee et al. reported that explainable models could classify different hemorrhage subtypes with high accuracy, while Wang et al. demonstrated similar results using RSNA datasets [14,15]. Yun et al. showed that AI support improved diagnostic accuracy, particularly among non-expert readers [13]. In more recent studies, Kang et al. also emphasized that AI-assisted evaluation may improve reader performance, especially in equivocal cases [20]. Therefore, the high kappa value observed in our study suggests that AI systems may provide meaningful prioritization and decision support in routine emergency radiology practice.

Subgroup analyses demonstrated that AI performance may vary according to the clinical context. The higher sensitivity observed in neurological presentations may be related to the greater likelihood of acute intraparenchymal or ventricular hemorrhage in these patients and the more targeted imaging indications. In contrast, the higher specificity observed in the trauma group suggests that the algorithm may be more effective in limiting false-positive results. However, in traumatic cases, small extra-axial hemorrhages, bone-related artifacts, and complex anatomical alterations may have contributed to reduced sensitivity. Similarly, Mabit et al. reported that AI systems achieved high sensitivity and specificity in trauma populations, although certain false-positive findings persisted [21]. In the scanner-integrated system study conducted by Kiefer et al., high negative predictive values were achieved, whereas the positive predictive value was relatively lower [22]. These findings suggest that, in trauma patients, AI may be more appropriately used as an adjunctive tool primarily focused on minimizing missed diagnoses.

In the shift-based analysis, it was noteworthy that although AI interpretation times remained stable, diagnostic performance declined during night shifts. This finding may be related less to algorithmic fatigue and more to the increased clinical complexity of patients imaged during nighttime hours and the greater burden of technical artifacts. Hanna et al. similarly reported an association between heavy shifts, increased workload, and interpretation errors [5]. Therefore, although AI systems may have the potential to reduce human fatigue, they cannot completely eliminate the challenges posed by the complex patient population encountered during night shifts.

The strong correlation observed between the AI system and the ABC/2 method in hemorrhage volume analysis indicates that AI-based volumetric assessment may be clinically applicable. Kothari et al. demonstrated that the ABC/2 method is a rapid and practical approach for hemorrhage volume estimation [23]. Gebel et al. reported that this method provided reliable results in intraparenchymal and subdural hematomas, while Won et al. demonstrated a high correlation between ABC/2 and volumetric analysis in subdural hematomas [24,25]. In contrast, Freeman et al. noted that the error margin of the ABC/2 method may increase in irregularly shaped hematomas [26]. Our findings suggest that AI-assisted volume measurement may provide practical support, particularly in routine clinical practice.

Overall, our findings indicate that AI may be more appropriately utilized not as an independent system replacing the radiologist in the diagnosis of intracranial hemorrhage, but rather as a decision-support tool that facilitates the early identification of critical cases and supports emergency imaging workflow. This interpretation is consistent with systematic reviews and real-world evidence reported in the literature [12,16,19,20,21,27]. Furthermore, the study by Kotovich et al., which demonstrated improved clinical outcomes following AI integration, suggests that such systems may contribute positively to patient management [28]. Of particular relevance to the current findings is a recent prospective multicenter diagnostic accuracy study by Khoruzhaya et al. (2025), which directly compared standalone AI performance with AI-assisted radiologist interpretation for intracranial hemorrhage detection on emergency brain CT [29]. That study demonstrated that while standalone AI achieved high sensitivity, the combination of AI assistance with radiologist interpretation yielded superior overall diagnostic accuracy and significantly reduced the miss rate for clinically significant hemorrhages compared to either approach alone. This evidence strongly supports the positioning of AI as a complementary tool within a radiologist-supervised workflow rather than as a replacement, which is consistent with the conclusions of the present study. The 14 false-negative cases (false-negative rate: 5.96%) identified in our study are of particular clinical relevance. The predominance of missed IVH and small-volume SDH cases highlights that the AI system’s sensitivity may be disproportionately reduced for low-attenuation, small-volume, or anatomically complex hemorrhages. Clinically, delayed detection of IVH or thin SDH may carry significant consequences, including delayed neurosurgical consultation and risk of neurological deterioration. These findings reinforce the position that AI should not be used as a standalone triage filter to exclude hemorrhage without physician oversight. The AI system’s role as a safety net for negative examinations should be understood within the context of a dual-read workflow—whereby AI-negative cases still receive timely radiologist review.

## 5. Limitations

This study has several limitations. First, its retrospective single-center design may limit the generalizability of the findings. Second, the relatively high prevalence of intracranial hemorrhage likely reflects the selected high-risk emergency neuroimaging population of a tertiary-care referral center and may have influenced diagnostic performance metrics. Third, external and multicenter validation analyses were not performed. Finally, clinical outcomes such as mortality, neurosurgical intervention, and treatment delay were not prospectively evaluated. Furthermore, the relatively high prevalence of intracranial hemorrhage in our cohort (62.7%) likely reflects the clinical referral pattern and selective imaging indications of a tertiary-care emergency center. This elevated prevalence may have inflated positive predictive values and should be considered when interpreting the results in settings with lower prior probability of hemorrhage. It is acknowledged that the high prevalence introduces a significant risk of selection bias; imaging indications were based on clinical judgment at a tertiary referral center rather than a consecutive unselected community emergency population, and therefore the diagnostic performance metrics reported here—particularly positive and negative predictive values—may not be directly applicable to lower-acuity or lower-prevalence settings. The absence of external or multicenter validation is a significant limitation. Dataset shift—defined as systematic differences in image characteristics, patient demographics, scanner protocols, and pathology prevalence between the training environment and deployment setting—is a well-recognized challenge in clinical AI deployment, and future prospective multicenter studies are essential to characterize the robustness of this AI system across diverse clinical environments. Additionally, the subgroup analyses by work shift were exploratory in nature, as the study was powered for the primary binary diagnostic performance analysis. Post hoc power analysis indicated that the night shift subgroup (*n* = 96) had approximately 74% power to detect the observed difference in sensitivity compared to the day shift reference at α = 0.05, which is below the conventionally accepted threshold of 80%; accordingly, these subgroup findings should be interpreted as hypothesis-generating. Finally, hemorrhage volume correlation was assessed against the ABC/2 formula rather than manual volumetric segmentation, which is the gold standard for volume measurement; future studies should compare AI-derived measurements against software-assisted or manual segmentation to more rigorously validate volumetric accuracy. A further and particularly important limitation relates to the black-box nature of the evaluated commercial AI system. Because the underlying model architecture, training data composition, class weighting strategies, and decision thresholds are proprietary and not publicly disclosed, it is not possible to independently audit the system for potential biases—including demographic biases related to age, sex, or ethnicity—or to fully characterize sources of model uncertainty. The absence of explainability outputs, such as saliency maps, class activation maps (CAMs), or lesion localization heatmaps, in the current clinical version of the software further limits the interpretability of AI-generated classifications. Without such tools, it is not possible to confirm that the model’s correct classifications are driven by clinically meaningful hemorrhage features rather than spurious correlations within the training data. These considerations reinforce the necessity of maintaining physician oversight in all AI-assisted diagnostic workflows and highlight the importance of regulatory frameworks requiring greater transparency from AI medical device manufacturers prior to clinical deployment. Additionally, the time-efficiency comparison in this study has an important methodological limitation: AI interpretation time (pure computational processing time) and radiologist interpretation time (total report turnaround time including queue delay, workload, dictation, and finalization) are not equivalent metrics. The observed 59 min difference therefore reflects system-level report availability time rather than a direct comparison of cognitive image reading speeds. Future studies should employ prospective designs that measure actual radiologist image reading time independently of administrative and workflow factors to enable a more valid comparison of AI and human interpretation speeds.

## 6. Conclusions

This study demonstrated that the artificial intelligence system provided high sensitivity and accuracy in detecting intracranial hemorrhage on emergency brain CT examinations, offered a significant reduction in interpretation time compared with radiologist evaluation, and showed strong agreement in both hemorrhage subtype classification and volume estimation. The findings suggest that the greatest strength of the system lies not in functioning as an autonomous reporting tool, but rather as a decision-support system that prioritizes critical cases, provides a safety net for negative examinations, and supports emergency imaging workflow, particularly under conditions of heavy workload. Trauma cases, night-shift evaluations, and mixed/ventricular hemorrhage patterns appear to represent the clinical contexts in which the system should be interpreted with greater caution.

## Figures and Tables

**Table 1:** Baseline Demographic and Clinical Characteristics of the Study Population.

Variables	Overall (*n* = 375)	Hemorrhage (+) (*n* = 235)	Hemorrhage (−) (*n* = 140)	*p*
Patients, *n*	375	235	140	-
Age, years, mean ± SD	52.8 ± 20.8	57.4 ± 19.8	45.2 ± 20.1	**<0.001 **
Sex—Male, *n* (%)	203 (54.1)	132 (56.2)	71 (50.7)	0.238
Triage Score, mean ± SD	2.9 ± 1.1	2.4 ± 0.9	3.1 ± 1.1	**<0.001**
**Presentation Type**				
Neurological, *n* (%)	210 (56.0)	152 (64.7)	58 (41.4)	**<0.001**
Trauma, *n* (%)	165 (44.0)	83 (35.3)	82 (58.6)	—
**Diagnosis Distribution (Reference standard)**				
Normal (No Hemorrhage), *n* (%)	140 (37.3)	—	—	—
Intraparenchymal Hemorrhage (IPH), *n* (%)	72 (19.2)	—	—	—
Subdural Hematoma (SDH), *n* (%)	58 (15.5)	—	—	—
IPH + Intraventricular Hemorrhage, *n* (%)	51 (13.6)	—	—	—
Intraventricular Hemorrhage (IVH), *n* (%)	26 (6.9)	—	—	—
Epidural Hematoma (EDH), *n* (%)	28 (7.5)	—	—	—
**Shift Distribution**				
Day shift (08:00–16:00), *n* (%)	166 (44.3)	—	—	—
Evening shift (16:00–24:00), *n* (%)	113 (30.1)	—	—	—
Night shift (00:00–08:00), *n* (%)	96 (25.6)	—	—	—

**Table 2:** Comparison of Interpretation Times Between AI System and Radiologist (Reference standard).

Parameter	AI System	Radiologist (Reference Standard)	Time Difference (RS − AI), min	Statistic	*p*
**Overall Interpretation Times **					
Mean ± SD (min)	11.9 ± 3.0	70.9 ± 25.4	59.0	W = 0.0	**<0.001**
Median (IQR) (min)	12.0 (9.6–14.0)	69.8 (50.9–89.6)	—	—	—
Min–Max (min)	4.6–18.6	28–115	—	—	—
**Interpretation Time by Shift (min)**					
Day shift (*n* = 166)	11.8	69.4	57.6	—	—
Evening shift (*n* = 113)	12.1	71.8	59.7	—	—
Night shift (*n* = 96)	11.9	72.0	60.1	H = 1.24	>0.05
Inter-shift comparison (Kruskal–Wallis)	—	—	—	H = 1.24	>0.05

AI: Artificial Intelligence; RS: Reference standard; SD: Standard Deviation; IQR: Interquartile Range; W: Wilcoxon signed-rank test statistic; H: Kruskal–Wallis test statistic. Wilcoxon signed-rank test (non-parametric, paired) was used for overall comparison. Statistically significant *p* values (*p* < 0.05).

**Table 3:** Binary Diagnostic Performance of the AI System (Hemorrhage vs. No Hemorrhage).

	RS: Hemorrhage (+)	RS: Hemorrhage (−)	Total
AI System: Hemorrhage (+)	TP = 221	FP = 17	238
AI System: Hemorrhage (−)	FN = 14	TN = 123	137
Total	235	140	375
**Performance Metric**	**Value (95% CI)**		
**Core Diagnostic Metrics**			
Sensitivity	94.0% (91.2–96.2)		
Specificity	87.9% (84.2–90.9)	-	
Positive Predictive Value (PPV)	92.9% (90.0–95.1)	-	
Negative Predictive Value (NPV)	89.8% (86.3–92.5)	-	
Accuracy	91.7% (88.6–94.1)	-	
F1 Score	0.935	-	
Matthews Correlation Coefficient (MCC)	0.823	-	
**Agreement and Curve Analysis**			
AUC (ROC Curve)	0.909 (0.875–0.939)	-	-
Cohen’s Kappa (Binary)	0.823	-	-
Positive Likelihood Ratio (+LR)	7.76	-	-
Negative Likelihood Ratio (−LR)	0.068		-

CI: Confidence Interval (Wilson method); TP: True Positive; FP: False Positive; TN: True Negative; FN: False Negative; AUC: Area Under the Curve; MCC: Matthews Correlation Coefficient; LR: Likelihood Ratio; RS: Reference standard; PPV: Positive Predictive Value; NPV: Negative Predictive Value. Bootstrap resampling (2000 iterations) was used for AUC confidence intervals.

**Table 4:** Category-Level Diagnostic Performance of the AI System.

Diagnosis Category	*n* (RS)	Correct AI *n* (%)	Sensitivity (95% CI), %	Specificity (95% CI), %	PPV (95% CI), %	Cohen’s κ
Normal (No-Hemorrhage)	140	130 (92.9)	92.9 (89.8–95.1)	90.6 (87.2–93.2)	89.1 (85.5–91.9)	0.841
Intraparenchymal Hemorrhage (IPH)	72	66 (91.7)	91.7 (88.5–94.1)	93.8 (90.9–95.8)	86.8 (83.0–89.9)	0.782
Subdural Hematoma (SDH)	58	53 (91.4)	87.9 (84.2–90.8)	92.4 (89.3–94.7)	83.6 (79.5–87.0)	0.761
IPH + Intraventricular Hemorrhage (IPH + IVH)	51	44 (86.3)	86.3 (82.4–89.4)	94.7 (91.9–96.5)	81.4 (77.2–85.0)	0.741
Intraventricular Hemorrhage (IVH)	26	22 (84.6)	84.6 (80.6–87.9)	96.1 (93.6–97.6)	78.6 (74.2–82.5)	0.712
Epidural Hematoma (EDH)	28	25 (89.3)	89.3 (85.8–92.0)	95.2 (92.5–96.9)	86.2 (82.3–89.3)	0.748
**Overall Categorical Agreement Summary **						
Exact Agreement, *n* (%)	338 (90.1%)	—	—	—	—	—
Partial Agreement, *n* (%)	1 (0.3%)	—	—	—	—	—
Disagreement, *n* (%)	36 (9.6%)	—	—	—	—	—
Weighted Kappa (quadratic)	κ = 0.892	—	—	—	—	—

CI: Confidence Interval (Wilson method); RS: Reference standard; PPV: Positive Predictive Value; IPH: Intraparenchymal Hemorrhage; IVH: Intraventricular Hemorrhage; SDH: Subdural Hematoma; EDH: Epidural Hematoma. One-vs-rest approach was applied for category-level metrics. Weighted Kappa: quadratic weighting; κ > 0.80 denotes almost perfect agreement.

**Table 5:** Subgroup Analysis by Presentation Type and Work Shift.

Subgroup	*n*	Sensitivity (95% CI), %	Specificity (95% CI), %	Accuracy, %	AUC	Cohen’s κ	*p* Value
**A. By Presentation Type **							
Neurological	210	94.8 (90.9–97.1)	86.7 (81.4–90.6)	91.4	0.952	0.843	Ref.
Trauma	165	87.1 (81.1–91.4)	94.2 (89.5–96.9)	90.3	0.901	0.798	**0.011**
**B. By Work Shift**							
Day shift (08:00–16:00)	166	94.8 (90.3–97.3)	91.2 (85.9–94.6)	93.4	0.962	0.856	Ref.
Evening shift (16:00–24:00)	113	93.3 (87.1–96.6)	89.7 (82.7–94.1)	92.0	0.941	0.831	**0.031**
Night shift (00:00–08:00)	96	91.4 (84.1–95.5)	87.1 (78.9–92.4)	89.6	0.908	0.789	**<0.001**

For subgroup A: Z-test for sensitivity and specificity comparison. For subgroup B: pairwise z-test vs. day shift reference; overall inter-shift Kruskal–Wallis *p* = 0.031. AUC: Area Under the ROC Curve; CI: Confidence Interval (Wilson method); κ: Cohen’s Kappa. Ref.: Reference group. Statistically significant *p* values (*p* < 0.05).

## Data Availability

The datasets generated and/or analyzed during the current study are available from the corresponding author upon reasonable request.

## References

[1] van Asch C.J., Luitse M.J., Rinkel G.J., van der Tweel I., Algra A., Klijn C.J. (2010). Incidence, case fatality, and functional outcome of intracerebral haemorrhage over time, according to age, sex, and ethnic origin: A systematic review and meta-analysis. Lancet Neurol..

[2] Feigin V.L., Lawes C.M., Bennett D.A., Barker-Collo S.L., Parag V. (2009). Worldwide stroke incidence and early case fatality reported in 56 population-based studies: A systematic review. Lancet Neurol..

[3] Smith E.E., Rosand J., Greenberg S.M. (2006). Imaging of hemorrhagic stroke. Magn. Reson. Imaging Clin. N. Am..

[4] Boland G.W., Guimaraes A.S., Mueller P.R. (2008). Radiology report turnaround: Expectations and solutions. Eur. Radiol..

[5] Hanna T.N., Lamoureux C., Krupinski E.A., Weber S., Johnson J.O. (2018). Effect of Shift, Schedule, and Volume on Interpretive Accuracy: A Retrospective Analysis of 2.9 Million Radiologic Examinations. Radiology.

[6] Hemphill J.C., Greenberg S.M., Anderson C.S., Becker K., Bendok B.R., Cushman M., Fung G.L., Goldstein J.N., Macdonald R.L., Mitchell P.H. (2015). Guidelines for the Management of Spontaneous Intracerebral Hemorrhage: A Guideline for Healthcare Professionals From the American Heart Association/American Stroke Association. Stroke.

[7] Litjens G., Kooi T., Bejnordi B.E., Setio A.A.A., Ciompi F., Ghafoorian M., van der Laak J.A.W.M., van Ginneken B., Sánchez C.I. (2017). A survey on deep learning in medical image analysis. Med. Image Anal..

[8] Chilamkurthy S., Ghosh R., Tanamala S., Biviji M., Campeau N.G., Venugopal V.K., Mahajan V., Rao P., Warier P. (2018). Deep learning algorithms for detection of critical findings in head CT scans: A retrospective study. Lancet.

[9] Arbabshirani M.R., Fornwalt B.K., Mongelluzzo G.J., Suever J.D., Geise B.D., Patel A.A., Moore G.J. (2018). Advanced machine learning in action: Identification of intracranial hemorrhage on computed tomography scans of the head with clinical workflow integration. npj Digit. Med..

[10] Titano J.J., Badgeley M., Schefflein J., Pain M., Su A., Cai M., Swinburne N., Zech J., Kim J., Bederson J. (2018). Automated deep-neural-network surveillance of cranial images for acute neurologic events. Nat. Med..

[11] Greenberg S.M., Ziai W.C., Cordonnier C., Dowlatshahi D., Francis B., Goldstein J.N., Hemphill J.C., Johnson R., Keigher K.M., Mack W.J. (2022). 2022 Guideline for the Management of Patients With Spontaneous Intracerebral Hemorrhage: A Guideline From the American Heart Association/American Stroke Association. Stroke.

[12] Savage C.H., Tanwar M., Elkassem A.A., Sturdivant A., Hamki O., Sotoudeh H., Sirineni G., Singhal A., Milner D., Jones J. (2024). Prospective Evaluation of Artificial Intelligence Triage of Intracranial Hemorrhage on Noncontrast Head CT Examinations. AJR Am. J. Roentgenol..

[13] Yun T.J., Choi J.W., Han M., Jung W.S., Choi S.H., Yoo R.-E., Hwang I.P. (2023). Deep learning based automatic detection algorithm for acute intracranial haemorrhage: A pivotal randomized clinical trial. npj Digit. Med..

[14] Lee H., Yune S., Mansouri M., Kim M., Tajmir S.H., Guerrier C.E., Ebert S.A., Pomerantz S.R., Romero J.M., Kamalian S. (2019). An explainable deep-learning algorithm for the detection of acute intracranial haemorrhage from small datasets. Nat. Biomed. Eng..

[15] Wang X., Shen T., Yang S., Lan J., Xu Y., Wang M., Zhang J., Han X. (2021). A deep learning algorithm for automatic detection and classification of acute intracranial hemorrhages in head CT scans. NeuroImage Clin..

[16] Daugaard Jørgensen M., Antulov R., Hess S., Lysdahlgaard S. (2022). Convolutional neural network performance compared to radiologists in detecting intracranial hemorrhage from brain computed tomography: A systematic review and meta-analysis. Eur. J. Radiol..

[17] Maghami M., Sattari S.A., Tahmasbi M., Panahi P., Mozafari J., Shirbandi K. (2023). Diagnostic test accuracy of machine learning algorithms for the detection intracranial hemorrhage: A systematic review and meta-analysis study. BioMed. Eng. OnLine.

[18] Karamian A., Seifi A. (2025). Diagnostic Accuracy of Deep Learning for Intracranial Hemorrhage Detection in Non-Contrast Brain CT Scans: A Systematic Review and Meta-Analysis. J. Clin. Med..

[19] Agarwal S., Wood D., Grzeda M., Suresh C., Din M., Cole J., Modat M., Booth T.C. (2023). Systematic Review of Artificial Intelligence for Abnormality Detection in High-volume Neuroimaging and Subgroup Meta-analysis for Intracranial Hemorrhage Detection. Clin. Neuroradiol..

[20] Kang D.W., Kim M., Park G.H., Kim Y.S., Han M.-K., Lee M., Kim D., Ryu W.-S., Jeong H.-G. (2025). Deep learning-assisted detection of intracranial hemorrhage: Validation and impact on reader performance. Neuroradiology.

[21] Mabit L., Lepoittevin M., Valls M., Thomas C., Guillevin R., Herpe G. (2025). Real-Life Performance of a Commercially Available AI Tool for Post-Traumatic Intracranial Hemorrhage Detection on CT Scans: A Supportive Tool. J. Clin. Med..

[22] Kiefer J., Kopp M., Ruettinger T., Heiss R., Wuest W., Amarteifio P., Stroebel A., Uder M., May M.S. (2023). Diagnostic Accuracy and Performance Analysis of a Scanner-Integrated Artificial Intelligence Model for the Detection of Intracranial Hemorrhages in a Traumatology Emergency Department. Bioengineering.

[23] Kothari R.U., Brott T., Broderick J.P., Barsan W.G., Sauerbeck L.R., Zuccarello M., Khoury J. (1996). The ABCs of measuring intracerebral hemorrhage volumes. Stroke.

[24] Gebel J.M., Sila C.A., Sloan M.A., Granger C.B., Weisenberger J.P., Green C.L., Topol E.J., Mahaffey K.W. (1998). Comparison of the ABC/2 estimation technique to computer-assisted volumetric analysis of intraparenchymal and subdural hematomas complicating the GUSTO-1 trial. Stroke.

[25] Won S.Y., Zagorcic A., Dubinski D., Quick-Weller J., Herrmann E., Seifert V., Konczalla J. (2018). Excellent accuracy of ABC/2 volume formula compared to computer-assisted volumetric analysis of subdural hematomas. PLoS ONE.

[26] Freeman W.D., Barrett K.M., Bestic J.M., Meschia J.F., Broderick D.F., Brott T.G. (2008). Computer-assisted volumetric analysis compared with ABC/2 method for assessing warfarin-related intracranial hemorrhage volumes. Neurocrit. Care.

[27] Nada A., Sayed A.A., Hamouda M., Tantawi M., Khan A., Alt A., Hassanein H., Sevim B.C., Altes T., Gaballah A. (2025). External validation and performance analysis of a deep learning-based model for the detection of intracranial hemorrhage. Neuroradiol. J..

[28] Kotovich D., Twig G., Itsekson-Hayosh Z., Klug M., Ben Simon A., Yaniv G., Konen E., Tau N., Raskin D., Chang P.J. (2023). The impact on clinical outcomes after 1 year of implementation of an artificial intelligence solution for the detection of intracranial hemorrhage. Int. J. Emerg. Med..

[29] Khoruzhaya A.N., Sakharova P.A., Arzamasov K.M., Kremneva E.I., Burenchev D.V., Erizhokov R.A., Omelyanskaya O.V., Vladzymyrskyy A.V., Vasilev Y.A. (2025). Standalone AI Versus AI-Assisted Radiologists in Emergency ICH Detection: A Prospective, Multicenter Diagnostic Accuracy Study. J. Clin. Med..

